# Acknowledgements to referees 2013

**DOI:** 10.1186/1129-2377-15-4

**Published:** 2014-01-28

**Authors:** Valerio De Angelis

**Affiliations:** 1 Department of Clinical and Molecular Medicine, Sapienza University of Rome, Italy

## Abstract

**Contributing reviewers:**

The quality of *The Journal of Headache and Pain* depends on the qualified and regular collaboration of renowned scientists, who devoted their time to constructively review the submitted articles.

We are indebted to the following experts who reviewed papers that completed the peer-reviewing process within 2013.

## 

A. Alberti

Perugia

M.A. Al-Jumah

Riyadh

A. Ambrosini

Pozzilli

A.P. Andreou

London

F. Antonaci

Pavia

G. Appendino

Novara

A. Arboix

Barcelona

A. Ashkenazi

Doylestown

M. Ashina

Copenhagen

S. Ashina

New York

Y.I.A. Ayzenberg

Bochum

H. Balaban

Sivas

R. Balasubramaniam

Perth

P. Barbanti

Rome

M. Bartolini

Ancona

P.A. Battistella

Padua

L. Bendtsen

Copenhagen

R. Benoliel

Jerusalem

A. Blumenfeld

Encinitas

P. Böckerman

Helsinki

A. Bozzao

Rome

F. Brighina

Palermo

D. Buse

New York

M.G. Buzzi

Rome

M. Carotenuto

Naples

R. Casale

Montescano

S. Cevoli

Bologna

C. Chittleborough

Adelaide

G. Coloprisco

Rome

G. Coppola

Latina

P. Cortelli

Bologna

M. de Tommaso

Bari

G. Della Marca

Rome

C. Di Lorenzo

Latina

V. Di Piero

Rome

A. Donnet

Paris

A. Dowson

London

A. Ducros

Paris

P.L. Durham

Springfield

G. Dussor

Tucson

M. Dux

Szeged

B. Edvardsson

Lund

A.K. Erdemoglu

Kirikkale

C. Fernández-de-Las-Peñas

Madrid

A. Ferrari

Modena

A. Finkel

Chapell Hill

E. Franzoni

Bologna

J.L. Fuh

Taipei

H. Göbel

Kiel

C. Gaul

Essen

G. Gentile

Rome

P. Geppetti

Florence

R. Gerwin

Bethesda

M.A. Giamberardino

Chieti

P.J. Goadsby

San Francisco

F. Granella

Parma

A.L. Guerrero

Valladolid

K. Hagen

Trondheim

A. Hershey

Cincinnati

M. Hirano

Nara

R. Howells

Exeter

S. Jafarian

Tehran

M.M. Jan

Jeddah

L.J. Jin

Shangai

T.P. Jürgens

Hamburg

M.A. Kabbouche

Cincinnati

N. Karli

Bursa

Z. Katsarava

Unna

E. Kaufman

Cleveland

A. Kocer

Düzce

K. Ikeda

Tokio

C. Lampl

Linz

M. Lantéri-Minet

Nice

J. Lawley

Bangor

R. Leira

Santiago de Compostela

M. Linde

Trondheim

C. Lundqvist

Lørenskog

M.L. Martin

Mountain Terrace

M. Matharu

London

F. Mazzilli

Rome

G. Mengistu

Addis Ababa

A. Negro

Rome

A. Ozge

Yenişehir

J. Ong

Chicago

A. Ortiz

San Antonio

A. Oterino

Santander

K. Paemeleire

Ghent

I.H. Pacheva

Plovdiv

J.A. Pareja

Alcorcón

P. Parisi

Rome

J. Pepe

Rome

B.L. Peterlin

Baltimore

M. Peters

Oxford

D. Pietrobon

Padua

L.A. Pini

Modena

M. Pompili

Rome

S.W. Powers

Cincinnati

P. Pozo-Rosich

Antofagasta

S. Prakash

Baroda

A. Purdy

Halifax

L.P. Queiroz

Florianopolis

K. Rabe

Essen

A. Raggi

Milan

D. Restuccia

Rome

F. Riederer

Zurich

X. Rodriguez

Santiago de Compostela

T.D. Rozen

Wilkes-Barre

M.B. Russell

Oslo

S. Sacco

L’Aquila

T. Sand

Trondheim

P. Sarchielli

Perugia

S. Seidel

Vienna

G. Serafini

Rome

Y. Shen

San Francisco

A. Sieminska

Gdansk

S. Silberstein

Philadelphia

S. Slater

Cincinnati

B.-W. Soong

San Francisco

K. Spiegelhalder

Freiburg

L.J. Stovner

Trondheim

A. Straube

Munich

M.L. Sumelahti

Tampere

O. Summ

Münster

C. Tassorelli

Pavia

G. Tedeschi

Naples

G. Terwindt

Leiden

D. Toni

Rome

P. Torelli

Parma

M. Trappolini

Rome

A. Truini

Rome

G. Tsivgoulis

Alexandroupolis

A. Tyagi

Glasgow

D. Valade

Paris

V. Villani

Rome

Y. Wang

Taipei

H.W. Schytz

Glostrup

C. Wöber

Vienna

C. Wöber-Bingöl

Vienna

T. Wolter

Freiburg

S.Y. Yu

Beijing

J.M. Zakrzewska

London

V. Ziaee

Tehran

